# Presence of Hypertension Is Reduced by Mediterranean Diet Adherence in All Individuals with a More Pronounced Effect in the Obese: The Hellenic National Nutrition and Health Survey (HNNHS)

**DOI:** 10.3390/nu12030853

**Published:** 2020-03-23

**Authors:** Emmanuella Magriplis, Demosthenes Panagiotakos, Ioannis Kyrou, Costas Tsioufis, Anastasia-Vasiliki Mitsopoulou, Dimitra Karageorgou, Ioannis Dimakopoulos, Ioanna Bakogianni, Michalis Chourdakis, Renata Micha, George Michas, Triantafyllia Ntouroupi, Sophia-Maria Tsaniklidou, Kostantina Argyri, Antonis Zampelas

**Affiliations:** 1 Department of Food Science and Human Nutrition, Agricultural University of Athens, Iera Odos 75, 118 55 Athens, Greece; emagriplis@eatsmart.gr (E.M.); av.mitsopoulou@gmail.com (A.-V.M.); dimi.karageorgou@gmail.com (D.K.); ioannis.dimakopoulos@gmail.com (I.D.); ioanna_bakogianni@hotmail.com (I.B.); Renata.Micha@tufts.edu (R.M.); gv.michas@gmail.com (G.M.); tdouroup@yahoo.gr (T.N.); smtsanik@hotmail.com (S.-M.T.); nargiri@gmail.com (K.A.); 2 Department of Nutrition and Dietetics, School of Health Science and Education Harokopio University, Athens, Eleftheriou Venizelou 70, 176 76 Athens, Greece; d.b.panagiotakos@usa.net (D.P.); kyrouj@gmail.com (I.K.); 3 Aston Medical Research Institute, Aston Medical School, Aston University, Birmingham B4 7ET, UK; 4 WISDEM, University Hospitals Coventry and Warwickshire NHS Trust, Coventry CV2 2DX, UK; 5 Warwick Medical School, University of Warwick, Coventry CV4 7AL, UK; 6 1st Department of Cardiology, Medical School, National and Kapodistrian University of Athens, Mikras Asias 75, 115 27 Athens, Greece; ktsioufis@gmail.com; 7 Friedman School of Nutrition Science and Policy, Tufts University, Boston, MA 02110, USA; 8 Laboratory of Hygiene, Social & Preventive Medicine and Medical Statistics, Department of Medicine, Faculty of Health Sciences, Aristotle University of Thessaloniki, University Campus, 54 124 Thessaloniki, Greece; mhourd@gapps.auth.gr; 9 Department of Cardiology, “Elpis” General Hospital of Athens, 106 75 Athens, Greece

**Keywords:** nutrition-health survey, hypertension prevalence, Mediterranean diet adherence, overweight and obesity, comorbidities, nutrition

## Abstract

Hypertension is a major risk of cardiovascular diseases. This study’s aim was to examine associations between hypertension and a priori known lifestyle risk factors, including weight status and Mediterranean diet adherence. The study included a representative sample of the adult population (N = 3775 (40.8% males)), from the Hellenic National Nutrition and Health Survey (HNNHS), which took place from September 2013 to May 2015. Demographic and anthropometric data were collected using validated questionnaires, and blood pressure (BP) measurements were performed for the two main metropolitan areas (N = 1040; 41.1%). Hypertension diagnosis was according to the International Classification of Diseases (ICD-10) guidelines. Weighted proportions, extended Mantel–Haenszel (M–H) analyses, and multiple logistic regressions (for the survey data) were performed. Mean systolic BP (SBP) and diastolic BP (DBP) were 118.6 mmHg and 72.2 mmHg respectively, with both values being higher in males compared to females in all age groups (*p* < 0.001). Study participants with hyperlipidemia or diabetes, and those overweight, were almost twice as likely to be hypertensives, with the odds increasing to 4 for those obese (*p* for all, < 0.05). Stricter Mediterranean diet adherence significantly decreased the likelihood of hypertension by 36% (OR: 0.64; 95% CI: 0.439, 0.943), and a significant interaction was found between Mediterranean diet adherence and weight status on hypertension. The presence of hypertension is clustered with comorbidities, but is significantly associated with modifiable risk factors, including Mediterranean diet and weight status, underlining the need for personalized medical nutritional treatment.

## 1. Introduction

Hypertension is a leading risk factor for cardiovascular (CVD) and chronic kidney diseases (CKD) [1,2], frequently remaining asymptomatic. Recent data have indicated trends for decreased systolic (SBP) [3,4] and diastolic (DBP) blood pressure [4] globally, mostly in middle- and high-income countries. However, these trends appear to vary significantly across regions and countries, depending on genetic and epigenetic predisposition, socioeconomic status, access to health services and multiple highly modifiable anthropometric and lifestyle factors (e.g., body weight, smoking, dietary patterns and physical activity level) [3,4]. As such, it is now well-accepted that these factors must be also assessed in health surveys evaluating hypertension status by region/country.

Overall, the prevalence of hypertension in adults over 18 years old is estimated at 30–45% [5], with age-standardized hypertension prevalence rates of 24% and 20% in men and women, respectively [4], and significant geographical variation [6]. Moreover, high blood pressure (BP) was estimated to account for 9.4 million deaths globally in 2010, with this detrimental impact increasing by 2.1 million compared to 1990 [5]. Of note, over 40% of worldwide deaths from major chronic noncommunicable diseases have been attributed to high BP, with over 63% of them due to the combined effect of high BP, increased body mass index (BMI) and elevated plasma glucose and cholesterol levels [2]. The Global Burden of Disease Study estimated that 22.4% and 20.7% of all male and female deaths, respectively, were attributable to poor dietary habits [7]. In addition, a large intervention study that used two Mediterranean dietary patterns, supplemented either with extra virgin olive oils or nuts, a pattern that characterizes the eating habits of Greeks in the past, found these diets to decrease SBP [8].

In Greece, over the past 20 years, cross-sectional studies have reported hypertension prevalence rates ranging between 13% and 27% [9]. Similar rates have been also reported by the ATTICA study, a sub-national study in the Attica region of Greece (2002–2012), which included a representative sample of rural and urban areas of this region of the country [10].

To address the need for up-to-date and generalizable data on the status of hypertension in Greece and its association with major risk factors, as well as Mediterranean diet adherence, the aim of the present study was to identify key characteristics and potential associations of hypertension (e.g., associations with lifestyle and sociodemographic factors) in a representative sample of Greek adults from the general population who participated in the Hellenic National Nutrition and Health Survey (HNNHS).

## 2. Materials & Methods

### 2.1. Study Design

The HNNHS was a cross-sectional observational survey, which was carried out from 2013 to 2015 and surveyed non-institutionalized individuals of all ages, living in Greece [11]. Study stratification was performed based on: (i) geographical density criteria by Greek region (7 regions); (ii) age group; and (iii) gender distribution, as provided by the Hellenic Statistical Authority. Households served as primary sampling units (PSU), with only one individual per age-group (0–19; 20–64; 65+) and per household being eligible for selection. Complete details of the study design, including inclusion/exclusion criteria, have been previously published [11].

In brief, a total of 4574 individuals consented to participate (men: 42.5% and women: 57.5%; 0–19 years old: 19%, 20–64 years old: 67%, and 65+ years old: 14%) in the HNNHS, with the sample distributed throughout Greece. Post hoc assessment resulted in a study power of 92% (for an effect size Odds Ratio [OR] = 1.2), accounting for a large population (N> 10,000). From the total HNNHS study sample, data from 3775 non-institutionalized adult (>19 years old) men and non-pregnant or lactating women (40.8% males) were analyzed for the purposes of the present study, in order to characterize hypertension status and associations in Greek adults of the general population. The survey included a standardized in-home interview and a physical examination for all individuals, and an additional medical examination (using a mobile center) for individuals residing in the two main metropolitan areas. A total of 1040 participants (from 2532 adults primarily recruited; 41%) consented to the latter. No differences in the age distribution were found between those measured and the total adult sample in male and female participants (*p* = 0.302 and *p* = 0.574 for females and males, respectively).

The HNNHS was approved by the Ethics Committee of the Department of Food Science and Human Nutrition of the Agricultural University of Athens and by the Hellenic Data Protection Authority (HDPA), and each study participant provided informed consent for the study.

### 2.2. Parameters Investigated

Interview-based questionnaires were used to obtain information on sociodemographic characteristics, medication history, and lifestyle status (e.g., smoking habits and physical activity level). Blood pressure status was also evaluated through medical history, and the International Classification of Diseases (ICD, 10th version) codes were recorded by experienced study clinicians.

#### 2.2.1. Sociodemographic Data

Data on age, sex, educational level and employment status were computed. Educational level was classified into 3 groups: low: elementary schooling or lower; medium: up to high-school education or technical college; and high: college or higher degree of education. Regarding employment status, individuals were categorized as employed, retired (on pension) or unemployed, with the latter defined as those on temporary or permanent dismissal.

#### 2.2.2. Lifestyle & Anthropometric Factors

In the context of this study, smoking, physical activity level and BMI were assessed. As such, study participants were classified as smokers (reporting daily or occasional smoking) or non-smokers. Physical activity was evaluated using the validated International Physical Activity Questionnaire (IPAQ) adapted for adults and elderly, with physical activity status defined as light, moderate or high based on the IPAQ score, as per guidelines [12]. Individuals scoring below the light activity level were categorized as sedentary. Body weight (kg) and height (m) were used to calculate BMI, given by the equation weight/height^2^ (kg/m^2^), and participants were categorized based on BMI as underweight (BMI < 18 kg/m^2^), normal weight (18 ≤ BMI < 25 kg/m^2^), overweight (25 ≤ BMI < 30 kg/m^2^), or obese (BMI ≥ 30 kg/m^2^).

#### 2.2.3. Blood Pressure Status and Measurement

Study participants were categorized as normotensive or hypertensive by experienced clinicians, based on ICD-10 diagnosis codes or if they had been diagnosed as hypertensive at least once in the past by a clinician, and/or if they were taking any antihypertensive medication.

Blood pressure measurements were performed according to European Society of Hypertension (ESH) and European Society of Cardiology (ESC) guidelines for accurate BP measurement [5], using validated and calibrated auscultatory devices (Omron Hem 907, Vernon Hills, IL, USA) and appropriately sized cuffs (small, medium and large cuff sizes).

BP measurements were taken with individuals rested for at least 5 min, seated with their back upright, and their arm well-supported at a 45° angle from the trunk at the heart level. For both SBP and DBP, the mean value was derived from three consecutive measurements taken on a single occasion, at least a minute apart. Based on these measurements, hypertension was defined as average SBP ≥ 140 mmHg, and/or DBP ≥ 90 mmHg [13]. Study participants with hypertension were further classified as having Stage-I (140 < SBP < 159 mmHg and/or 90 < DBP < 99 mmHg) or Stage-II (SBP ≥ 160 mmHg and/or DBP ≥ 100 mmHg) hypertension. The percent of the study population that would be classified as having hypertension based on the 2017 American College of Cardiology/American Heart Association (ACC/AHA) clinical practice recommendations for the evaluation of high BP in adults [14] (SBP ≥ 130 mmHg and/or DBP ≥ 80 mmHg) was also estimated.

#### 2.2.4. Additional Parameters Measured

Two 24-h recalls were used to derive participants’ total sodium intake from food and Mediterranean diet adherence. More details on the methodology of the recall have been published elsewhere [11]. In summary, the two recalls were interview-based using validated-by-age-group food atlases, and were performed on different non-consecutive days, 8 to 20 days apart. Sodium intake was categorized to above and below 1500 mg per day, since sodium content was for food only (not salt added during cooking or table salt). Adherence to the Mediterranean diet was assessed using the MedDiet Score (range 0–55), as previously derived and validated by Panagiotakos et al. [15]. Briefly, this pattern consists of 11 food groups; fruits and vegetables, olive oil, non-refined cereals–carbohydrates (daily intake), dairy products (in moderation), fish and pulses/legumes, poultry, olives, and nuts (weekly intake), red meat and byproducts (monthly), and alcohol (in moderation). Specific food groups and equivalent mean daily or weekly intake (from the two 24 hr-recalls) was derived for each food group for all participants, and scores were applied accordingly. The scores ranged from 0 to 55, with higher score values indicating better adherence to the Mediterranean dietary pattern and lower scores suggesting a “Western-type” dietary pattern. According to Panagiotakos et al., an 11-unit increase in the MedDiet Score is associated with a 37% odds reduction in acute coronary events; therefore, the HNNHS participants were initially categorized into five MedDiet Score groups (i.e., MedDiet Score: 0–11; 12–22; 23–34; 35–44 and 45–55). Since limited numbers of study participants received either very low or very high MedDiet scores, and in order to achieve power in the preformed analyses, the final MedDiet groups were 0–22; 23–34; and 35–55, and a score ≥23 was applied as the cut-off for the logistic regression models of this study.

Hyperlipidemia and diabetes status were also estimated from medical history, and ICD-10 version codes were recorded by experienced study health professionals (hyperlipidemia: based on diagnosis of hypercholesterolemia and/or hypertriglyceridemia, or on antihypertensive medication; type 2 diabetes mellitus: based on diagnosis or on anti-diabetes medication).

### 2.3. Data Analysis

To account for the survey’s complex design, and in order to better reflect known strata in the population, a weighted average of the population (total population/sampled population) was obtained, accounting for the Primary Sample Unit (PSU). In detail, post-stratification weights were calculated by 10-year age group (weight 1) and sex (weight 2), based on the 2011 census. Household was set as the PSU to account for similarity within cluster (PSU = 2927), and the finite population correction (FPC) was used to calculate the standard error (SE) of the estimate(s), as the population size of the census had over 10,000 elements (N = 10,816,286).

### 2.4. Statistical Methodology

Unless stated otherwise, continuous variables are presented as mean ± standard deviation (SD), whilst categorical variables are presented as absolute numbers and frequencies (%). To account for sex differences, all analyses were stratified by sex. A Student’s t-test was used to compare sex differences and a chi-square test for continuous and categorical data, respectively. Weighted proportions and linearized standard errors (SE) were reported, to account for the complexity of the study’s design (svy:), and linear combinations of estimators (lincom) were used to examine the differences per age group and/or sex and/or area. Extended Mantel–Haenszel (M–H) Statistics (otherwise known as Cochran M–H) were performed by sex, to examine linear trends between hypertension percentage and age groups (p for trend). Multiple logistic regression, for survey data, was used to examine the odds of being hypertensive by age, sex, BMI category, sodium intake, and various sociodemographic and lifestyle variables (i.e., education and activity level, respectively), with corresponding 95% confidence intervals (95% CI) and linearized SE. Each factor was first examined separately in a simple age- and sex-adjusted model. Factors found to have a significant effect, or those with an accepted a priori effect on hypertension odds, were included in the final logistic regression model to decrease potential confounding. A survey-specific logistic regression model (with linearized standard errors (SE)) was derived to assess the probable effect of significant crude predictive variables, as well as those a priori known, on the likelihood of hypertension. A sensitivity test for the model was performed using linktest and receiver operating characteristics (ROC). All p-value estimates were based on two-sided tests. The STATA 14.0 (StataCorp LLC, College Station, TX, USA) statistical package was used for the analysis.

## 3. Results

Table 1 presents selected key sociodemographic, anthropometric and lifestyle characteristics of all adult participants of the HNNHS, both in total and stratified by sex. All parameters other than hypertension prevalence (16.2% and 16.9% in males and females, respectively) and other metabolic-related comorbidities (dyslipidemia and diabetes) significantly differed between male and female participants, with more males being sedentary, smokers, overweight and obese, whereas a greater percent of females achieved a higher MedDiet score (*p* = 0.004) and had a university degree (*p* for all, excluding MedDiet score, <0.001; Table 1).

**Figure 1:**
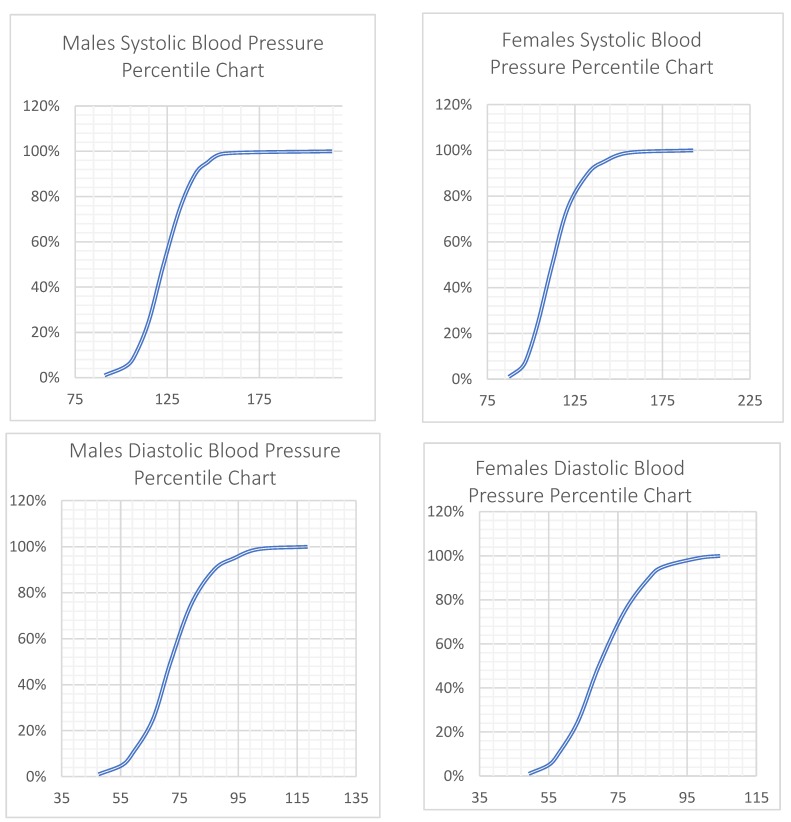
Blood Pressure Percentile Charts for males and females.

**Figure 2:**
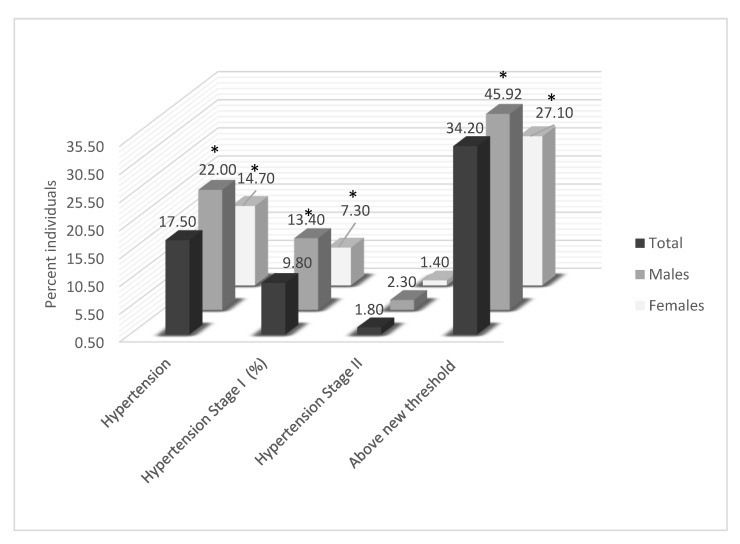
Prevalence of total hypertension; Stage I, Stage II and percent above new recommended guidelines, by sex. * Significant sex differences, *p* < 0.05. Total hypertension: defined as all stages including normotensives on anti-hypertensive medication. Stage I hypertension: % hypertensives with SBP > 140 and/or DBP > 90; Stage II hypertension: % hypertensives with SBP>160 and/or DBP>100; Above new threshold: % individuals with SBP > 130 and/or DBP > 80, as per new American College of Cardiology/American Heart Association (ACC/AHA) guidelines, in 2017.

In the adult study participants of the two major metropolitan areas of Greece (Attiki and Central Macedonia), the measured mean SBP and DBP were 118.6 (15.3) mmHg and 72.2 (10.6) mmHg, respectively (Table 2), with mean values being higher in males compared to females within all age groups (Table 2), as can be seen in the percentile charts in Figure 1 as well. The mean values for both SBP and DBP significantly differed by age within the female group of study participants, whereas only DBP differed in males, with a linear trend found in all cases (*p* for all <0.001; Table 2). Percentages of measured hypertension prevalence and categorization with stage-I or stage-II hypertension, and of participants with hypertension based on the 2017 ACC/AHA cut-off values, are also shown in Figure 1. Of the study participants with measured BP, 17.5% were categorized as having hypertension based on currently applied cut-offs, while 34.2% were so categorized based on the 2017 ACC/AHA recommended thresholds. In total, a higher proportion of males were hypertensive than females for both cut-offs (22.0% vs. 14.7%, *p* = 0.003; and 45.9% vs. 27.1, *p* = 0.002; Figure 2).

**Figure 3:**
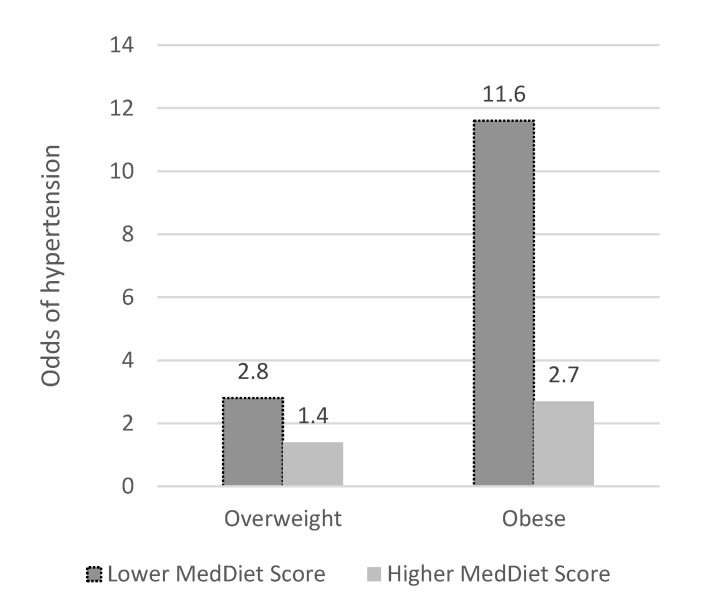
Odds of hypertension among overweight and obese participants compared to healthy weight, by MedDiet score. Chi square test for differences *p* < 0.001, Results based on multiple logistic regression: Fully adjusted for age, sex, sodium intake (>1500 mg/day vs. ≤1500 mg/day), employment status, educational and activity level, hyperlipidemia (presence of high cholesterol and/or triglycerides), and diabetes; Lower MedDiet Score <23; Higher MedDiet Score ≥23.

The logistic regression model resulted in a 0.8843 ROC, a value that represents high sensitivity. Based on the simple (i.e., age- and sex-adjusted) logistic regression analysis for each hypertension risk factor (e.g., weight status, dyslipidemia, diabetes, sodium/potassium intake, and Mediterranean diet adherence), the odds of being hypertensive were 3.6 times higher for overweight and seven times higher for obese individuals, compared to normal weight individuals. This remained significant in the fully adjusted model, with the effect being somewhat diluted, to two times and almost four times higher, respectively (Table 3). On the other hand, stricter adherence to the Mediterranean diet, as documented by a cut-off MedDiet Score >23, decreased the odds of being hypertensive (OR: 0.64; 95%CI: 0.439, 0.943; Table 3). A significant interaction was also found between Mediterranean diet adherence and weight status in the adjusted model. A higher MedDiet Score decreased the odds of being hypertensive by 36% and reached 60% when the interaction term (weight status) was added (Table 3). The odds of being hypertensive were significantly reduced from 4.9 (1.95; *p* < 0.001) to 2.45 (0.88; *p* = 0.12) for overweight and obese individuals with higher compared to lower Mediterranean diet adherence scores (data not shown). Hypertension was also associated with presence of hyperlipidemia and type 2 diabetes (*p* for all, <0.05). Individuals with a higher educational and physical activity level seemed to have lower odds of being hypertensive in the crude analysis; however, this did not remain significant in the fully adjusted model. Sex, smoking status, employment status and sodium intake showed no evidence of association. In Figure 3, the odds of hypertension by MedDiet score are shown, where, other than the total overweight and obese individuals seen in the model, a significantly lower hypertension likelihood was observed in overweight and obese individuals who achieved a higher MedDiet score *p* < 0.001).

## 4. Discussion

The main outcome of the present study was that hypertension was highly associated with weight status, with dietary habits modifying this association. Hypertension prevalence in all adults increased by 1% per year, according to the model used and as depicted by the linear significant increase in mean SBP and DBP values with age. Finally, the presence of hyperlipidemia and diabetes increased the likelihood of hypertension.

Mean SBP and DBP in all age groups were normal, but one in three males between 40 and 59 years, and over half of the male and female population over 60 years, had hypertension. According to the ESH–ESC guidelines [16], the estimated prevalence of hypertension was found to be 16.6% among adults throughout Greece [17], but when the new AHA cut-offs were used [14], the prevalence almost doubled (from 17.5% to 34%), both estimates suggesting that many Greek adults are at high risk of hypertension. Although the new cut-offs from ACC/AHA have not been adopted by ESC and ESH, these societies state that treatment via lifestyle modification and/or medication should aim to lower BP to <140/90 mmHg in all patients, with ideal target levels being 130/80 mmHg, in contrast to the 2013 European guidelines, being <140/90 mmHg in all and 140–150 mmHg for older patients (65–80 years) [16]. In addition, young, normal weight individuals were less likely to have been diagnosed with hypertension, while having dyslipidemia as a comorbidity was also more likely.

In Attiki alone, the prevalence of hypertension was 20% (specific results not shown) compared to the 30% reported in the ATTICA study [10]. In total, prevalence was 17.5%, compared to means of 16% reported by Pitsavos et al., [18] and 31% reported by the HYPERTEN–SHELL study [19]. Although we could infer that hypertension prevalence greatly varies between these studies, these cannot be directly compared due to study methodological differences (national vs. sub-national), whilst in the case of HYPERTEN–SHELL study, the population obtained was from those enrolled in various health centers. 

Although mean SBP and DBP have been reported as higher in European countries (136/83 mmHg) than in North America (127/77 mmHg) [20], in Greece, according to the HNNHS study, mean BP was closer to the North American values, with an average of 118.6/72.2 mmHg in adults over 20 years. Mean SBP and DBP were higher in males than females, with differences being more prominent in the younger age groups (less than 60 years), in accordance with most countries’ recent BP trends and data from the National Health and Nutrition Examination Survey (NHANES) III [4,14,21].

Results from observational studies have shown that BP progressively increases with age, adding to the long-term population burden of hypertension [14,22], and underlying the need for detecting hypertension early in life. It is well documented that excessive weight is associated with hypertension [23,24], with results from HNNHS showing that hypertension likelihood is almost two times greater in overweight and four times greater in obese individuals, irrespective of sex. This is also highlighted by a recent meta-analysis reporting that a 5 kg weight reduction was associated with a 4.4 and 3.6 mmHg decrease in SBP and DBP, respectively [25]. Mediterranean diet adherence decreased the likelihood of hypertension in accordance with other research findings [26] and was found to modify the association between obesity and hypertension. Obese participants that reported a higher adherence to the Mediterranean diet, although still at greater risk, had a two-fold lower likelihood of being hypertensive compared to obese participants with very low adherence. The Mediterranean diet has been shown to reduce CVD events [27,28] and improve surrogates of CVD, such as inflammatory markers [29], which can partly explain the moderating effect shown in this study. Furthermore, a recent summary of a prospective cohort study which specifically focused on Mediterranean diet and chronic disease reported that Mediterranean diet adherence mitigated the harmful effects of overweight and obesity on CVD risk [28]. It has also been shown by a randomized controlled trial that overweight or obese participants with untreated hypertension who were enrolled in a Mediterranean diet intervention group had a significant reduction in mean change from baseline in SBP and DBP, compared with controls at 4 months [30]. These findings help explain the moderating effect of Mediterranean diet adherence on weight status shown in this study and emphasize the need to assess lifestyle risk factors and establish more personalized medical nutritional therapies.

Both the very high prevalence of overweight and obesity found by HNNHS among Greek adults (32% overweight and 15.5% obesity), along with the increasing trend since the 1980s [31], and the decrease in Mediterranean diet adherence, emphasize the need for lifestyle changes in Greece. Hypertension was also associated with two other major CVD comorbidities, i.e., type 2 diabetes and hyperlipidemia [11] that prevail in Greece, as has been reported by others [18,32].

Other well-accepted risk factors, including smoking, sodium intake and physical activity [10,33,34] were not significantly associated with hypertension in the present study population. This may be due to the cross-sectional nature of this study, since lifestyle modifications that are observed upon diagnosis cannot be accounted for. It has been reported that 55% of young adults receive education upon being diagnosed with hypertension [35]. Additionally, sodium has been found to linearly increase BP in a dose–response relationship, with the effects being stronger in older individuals and among those with established hypertension [36]; however, this study included sodium intake from food only, and not the additional sodium (salt) added during cooking or eating, since this cannot be measured accurately in a large cross-sectional study, which may also partly explain the null finding. HNNHS did not evaluate total sodium intake from food and salt added during preparation or table salt, since the format of the study did not permit urine collection and analysis, which is required to accurately assess total salt intake. However, due to the latest Automated Multipass method used for the 24-h recalls, sodium from all food (including beverages) consumed was calculated and a cutoff of 1500 mg/day was set for further hypertension likelihood analysis. This was performed since the Mediterranean diet is naturally low in sodium due to its low degree of processing, unlike processed foods (a term that covers all foods that have undergone manufacturing methods, including convenience foods and products like bread, cheese and meat products) [37]. Overall only 10–12% of sodium occurs naturally in food and 75–80% of the salt in the U.S and in many European countries (such as the UK and Finland) comes from processed food [38], not from salt added during preparation or prior to consumption (table salt), and considering that the average current dietary salt intake is estimated between 9 and 12 g per day in Western countries and 7 to 13 g per day among adults in most European countries [39], the 1500 mg cutoff represents 75% of the 2000 mg WHO daily sodium target or 65% of the 2300 mg ESC target (percentages in the range of nutritional guidelines considering previous reports). In addition to the need for evaluating sodium in food, a recent study by Hasenegger et al., suggested that salt reduction can only be achieved in cooperation with food producers by the reduction of salt in processed foods, since the main other contributors to salt intake were daily consumed foods: mainly cereals and cereal products, meat and meat products, and dairy products [40].

### Limitations

Certain limitations of the present study should be acknowledged. For instance, accurate BP measurements were obtained from a study subsample, although no significant differences were found in age and sex distribution with the total study population. Furthermore, hypertension was diagnosed from the average measurements on a single occasion, potentially leading to overestimated hypertension prevalence [14]. However, this is common practice in epidemiological and population-based studies [41], making the results of this study comparable to relevant findings from the available literature. It could also be argued that 24-h recalls are not representative of usual intake, although they remain the gold standard along with medical history (as performed in this study) for obtaining accurate dietary intake and assessing sodium intake (food frequency questionnaires are inappropriate in this case). The 24-h recalls in the present study were interview based and performed 8–20 days apart for each participant, in order to give an estimation of dietary habits [16]. Additionally, 24-h recalls were deemed the appropriate method of dietary assessment, since blood pressure is readily affected by diet on a daily basis. This has been evaluated and reported by Domenech et al., [42] who studied a population that included 84% hypertensives and showed significant effects of the Mediterranean diet on blood pressure. These results were from diet advice alone, irrespective of sodium content and physical activity. Finally, the cross-sectional nature of this study does not allow causal associations, but only inferences.

## 5. Conclusions

The results of the present study emphasize the need for personalized lifestyle interventions aiming to decrease the risk of hypertension in all individuals, especially in those overweight and obese. Comorbidities such as hyperlipidemia and type 2 diabetes cluster with hypertension, further increasing the risk for CVD. As such, specifically addressing a major modifiable factor, i.e., dietary habits, by promoting adherence to the Mediterranean diet, which may have a beneficial impact on all these CVD risk factors, can significantly decrease the likelihood of hypertension in all participants, including overweight/obese individuals.

## Figures and Tables

**Table 1:** Demographic characteristics of the HNNHS study’s adult participants by sex.

	Total (N = 3775)	Males (N = 1541)	Females (N = 2234)	*p* Value for Sex Differences
Age (years), mean (sd)	43.6 (18.7)	43.1 (18,4)	44.0 (18.8)	**<0.001**
Weight (kg), mean (sd)	73.2 (15.5)	82.8 (13.6)	66.7 (13.2)	**<0.001**
Weight Status, *n* (%)				
Healthy Weight	1906 (52.3)	625 (41.8)	1281 (59.7)	**<0.001**
Overweight	1159 (31.8)	623 (41.6)	536 (25.0)	
Obese	577 (15.8)	248 (16.6)	329 (15.3)	
MedDiet Score, mean (sd)	28.7 (6.5)	28.1 (6.7)	29.0 (6.3)	**<0.001**
MedDiet Score, status, *n* (%)				**0.004**
0–22	616 (17.1)	286 (19.5)	330 (15.5)	
23–34	2219 (61.5)	896 (61.0)	1323 (62.2)	
35–55	771 (21.4	288 (19.6)	473 (22.3)	
Educational Status (level), *n* (%)				**<0.001**
Low	468 (12.4)	153 (9.9)	315 (14.1)	
Medium	1377 (36.6)	626 (40.7)	751 (33.7)	
High	1921 (51.0)	758 (49.3)	1163 (52.2)	
Smoking status, *n* (%)				**<0.001**
Non-smokers	2510 (66.5)	959 (62.2)	1551 (69.4)	
Smoker (all)	1265 (33.5)	582 (37.8)	683 (30.6)	
Physical activity status, *n* (%)				**<0.001**
Sedentary	286 (7.8)	136 (9.0)	150 (6.9)	
Light	515 (14.0)	233 (15.4)	282 (13.0)	
Moderate	1415 (38.5)	527 (34.8)	888 (41.0)	
Active	1463 (39.8)	617 (40.8)	846 (39.1)	
Hypertension, %(SE)	16.6 (0.01)	16.2 (0.01)	16.9 (0.01)	0.521
Other comorbidities, %(SE)				
Dyslipidemia *	20.2 (0.01)	19.3 (0.01)	20.9 (0.01)	0.208
Diabetes *	4.4 (0.0)	4.8 (0.01)	4.1 (0.00)	0.336

Significant at *p* < 0.05; * weighted proportions by population, age and sex distribution.

**Table 2:** Percentage distribution of blood pressure level in the adult Greek population by sex and age group.

		Age Group				Age Group			
	Total	20–39	40–59	60+		20–39	40–59	60+	
		Males			*p*-Value ^1^	Females			*p*-Value ^1^
Mean SBP, mean (SD)	118.6 (15.3)	124.8 (10.6)	126.9 (13.9)	128.9 (19.6)	0.082 *	109.3 (10.2) ^a,c^	116.8 (15.8) ^a,b^	126.2 (19.1) ^b,c^	<0.001 *
Mean DBP, mean (SE)	72.2 (10.6)	72.3 (10.1) ^a^	78.5 (10.8) ^a^	76.1 (11.1)	<0.001 *	68.6 (9.7) ^a,c^	73.4 (10.8) ^a^	72.2 (10.3) ^c^	<0.001 *
Total measured Hypertension ^2^, %	17.5	10.5	30.6	54.6	<0.001 *	2.5	15.2	55.8	<0.001 *

^1^ Statistical difference between age groups for each sex, at *p* < 0.05, using oneway ANOVA and Tukey test; ^a,b,c^: denotes statistical differences by age-group. * Significant *p* for trend, following Extended Mantel–Haenszel (M–H) statistics; Same superscript indicates significant difference; Total hypertension, meaning all stages including normotensives on anti-hypertensive medication.^2^ Based on mean of three measurements.

**Table 3:** Simple and multiple logistic regression analyses, evaluating the odds of hypertension by socio-demographic, lifestyle and biological factors.

	Odds Ratio (SE) *	95% Confidence Interval	Odds Ratio (SE) *	95% Confidence Interval
	Simple Logistic Regression ^1^		Final Model ^2^	
**Age (per 1 year)**	**1.1 (0.01)**	**1.089, 1.107**	**1.1 (0.01)**	**1.072, 1.102**
Females vs. Males	1.1 (0.13)	0.850, 1.236	0.92 (0.13)	0.694, 1.213
Smokers vs. non-smokers	1.02 (0.06)	0.894, 1.164	1.04 (0.08)	0.892, 1.205
**Educational level**				
Up to 6 years	base	-	base	
7–12 years	**0.15 (0.02)**	**0.111, 0.190**	0.92 (0.90)	0.756, 1.112
>12 years	**0.09 (0.01)**	**0.071, 0.122**	1.01 (0.15)	0.762, 1.340
**Weight Status**				
Healthy weight	base	-	base	-
**Overweight**	**3.60 (0.48)**	**2.770, 4.677**	**1.89 (0.33)**	**1.342, 2.679**
**Obese**	**7.06 (1.03)**	**5.305, 9.387**	**3.79 (0.71)**	**2.264, 5.481**
**MedDiet score (≥23 compared to <23)**	1.25 (0.19)	0.930, 1.681	**0.64 (0.13)**	**0.439, 0.943**
**Weight status × MedDiet Score (>23 compared to <23) ^3^**	0.51 (0.20)	0.235, 1.096	**0.40 (0.17)**	**0.170, 0.937**
Employment status				
Employed	base	-	base	
Unemployed	1.01 (0.19)	0.703, 1.461	0.98 (0.20)	0.655, 1.463
Pension	1.10 (0.22)	0.746, 1.634	1.11 (0.24)	0.723, 1.709
Physical activity level				
Sedentary	base	-	base	-
Low	**0.43 (0.09)**	**0.286, 0.645**	1.03 (0.32)	0.560, 1.911
Medium	**0.44 (0.08)**	**0.314, 0.623**	0.93 (0.26)	0.540, 1.607
High	**0.37 (0.07)**	**0.258, 0.526**	1.01 (0.28)	0.586, 1.745
Sodium intake (>1500 mg/day vs. <1500 mg/day)	1.04 (0.14)	0.802, 1.355	1.01 (0.15)	0.762, 1.340
**Hyperlipidemia (presence vs. non-presence)**	**5.33 (0.58)**	**4.318, 6.582**	**2.30 (0.33)**	**1.729, 3.055**
**Diabetes (presence vs. non-presence)**	**7.0 (1.26)**	**4.887, 9.902**	**1.78 (0.47)**	**1.057, 2.004**

^1^ Simple logistic regression: adjusted only for age, sex; ^2^ Multiple logistic regression: fully adjusted for age, sex, sodium intake (>1500 mg/day vs. ≤1500 mg/day), weight status (weight category) employment status, educational and activity level, hyperlipidemia (presence of high cholesterol and/or triglycerides), and diabetes. * linearized SE reported, due to survey data. ^3^ Interaction term between MedDiet and weight status (healthy weight with MedDiet adherence, compared to overweight and obese).

## Data Availability

Raw data were generated at [Agricultural University of Athens]. Derived data supporting the findings of this study are available from the corresponding author [AZ] on request.
